# A diagnostic tool for people with lumbar instability: a criterion-related validity study

**DOI:** 10.1186/s12891-021-04854-w

**Published:** 2021-11-23

**Authors:** Thiwaphon Chatprem, Rungthip Puntumetakul, Jaturat Kanpittaya, James Selfe, Gillian Yeowell

**Affiliations:** 1 Faculty of Associated Medical Sciences, Khon Kaen University, Khon Kaen, Thailand; 2 Research Center in Back, Neck, Other Joint Pain and Human Performance (BNOJPH), Faculty of Associated Medical Sciences, Khon Kaen University, Khon Kaen, 40002 Thailand; 3 The Thailand Research Fund (TRF), Bangkok, Thailand; 4 Department of Radiology, Faculty of Medicine, Khon Kaen University, Khon Kaen, Thailand; 5 Department of Health Professions, Faculty of Health and Education, Manchester Metropolitan University, Manchester, UK

**Keywords:** Instability test, Diagnostic, X-ray examination

## Abstract

**Background:**

Several clinical tests used to identify patients with lumbar instability have reported diagnostic accuracy in separate studies with conflicting results. To augment the diagnostic process, tests that are better able to identify lumbar instability suitable for use in the clinical setting are required. The aim of this study was to identify the probability to diagnose patients with lumbar instability, using x-ray imaging as the reference standard.

**Methods:**

This study was a cross-sectional, diagnostic validity study. One hundred forty participants with chronic low back pain underwent an x-ray assessment and 14 clinical examinations. Data were analysed using multivariate regression methods to determine which clinical tests were most diagnostic for lumbar instability when they were applied together.

**Results:**

Eighteen (12.85%) participants had radiological lumbar instability. Three clinical tests i) interspinous gap change during flexion-extension, ii) passive accessory intervertebral movement tests, iii) posterior shear test demonstrated an ability to diagnose lumbar instability of 67% when they were all positive. At this probability threshold, sensitivity, specificity, positive likelihood ratio (+LR), and negative likelihood ratio (−LR) were 5.56, 99.18%, 6.78, and 0.95.

**Conclusions:**

These 3 clinical tests could be useful in identifying patients with lumbar instability in the general community. These three tests are simple to perform by physical therapists, reliable to use in a clinical setting, and safe for patients. We recommend physical therapists use these three tests to assess patients who are suspected of having lumbar instability, in the absence of an x-ray assessment, to receive appropriate targeted intervention or referral for further investigation.

**Trial registration:**

Thai Clinial Trial Registry (TCTR 20180820001; 19th August 2018).

**Supplementary Information:**

The online version contains supplementary material available at 10.1186/s12891-021-04854-w.

## Background

Lumbar spinal stability is the ability of the lumbar spine to tolerate displacement during normal physiological postures and loads without the generation of nociceptive stimuli [1, 2]. Lumbar spine stability is achieved through the combination of three control subsystems; passive, active and neural control [2]. When at least one of these subsystems is compromised, lumbar spine movement can become abnormal [3], which can be detected through qualitative and quantitative assessments [4], such as self-reporting of symptoms and radiographic techniques [5, 6].

Lumbar instability has been identified in up to 57% of patients with chronic low back pain (CLBP) via X-ray [7]. The incidence rate of clinical lumbar instability in patients with CLBP is 13-46% [8, 9]. Lumbar instability can lead to pain, functional limitations and reduced quality of life [10–12], as well as further develop into spondylolisthesis in certain cases, which may require surgical treatment [1, 13]. Therefore, the prompt detection of lumbar instability is crucial to preventing disease progression or inhibiting adverse effects. Appropriate conservative treatment, such as exercise stabilization, which focuses on deep trunk muscle training, can improve or delay the development of lumbar instability [14–17]. In order to identify patients who can benefit from specific therapy, clinical decision-making is based on the accuracy of clinical examinations [13, 18, 19].

Flexion-extension X-ray is used to identify lumbar instability [1, 19]. However, it can only detect a loss of integrity in the passive subsystem, which is characterized by the excessive movement of one vertebra on another [19]. Additional limitations of X-ray-based diagnosis include the administration cost, accessibility, time cost and radiation exposure [13, 20]. A variety of clinical tests have been introduced to diagnose lumbar instability. Previous studies have investigated the diagnostic accuracy of several of these tests by comparing them to X-rays [7, 19, 21–24]. Treatment-based classification has been used to identify patients likely to benefit from stabilization exercises [25]. Previous studies have reported that the accuracy among tests varied widely, with sensitivity values of 5-84% and specificity values of 3-100% [7, 21–24]. Therefore, drawing any conclusions about the superiority of one test over another is difficult. To the best of our knowledge, diagnostic accuracy has so far been reported for 14 lumbar instability tests in separate studies with conflicting results [7, 21–25]. However, in a clinical setting, the physical therapist cannot perform 14 clinical tests, due to time constraints. Therefore, in order to improve the diagnostic process, the tests that are better able to identify lumbar instability and are suitable for the average clinical setting need to be determined. This study intends to investigate the predictive probability of the 14 instability tests to assess which tests are most suitable.

Two previous studies have reported the use of multivariate regression analysis in clinical examinations for the diagnosis of lumbar instability [7, 25]. The first study reported hypermobility in the passive accessory intervertebral movements test and a lumbar flexion range of > 53°. These two tests exhibited a high specificity of 98.0% and a positive likelihood ratio (+LR) of 12.8 [7]. The second study reported a specificity of 86.0% and a + LR of 4.0 when at least three of the following criteria were met: prone instability test, positive aberrant motion test, average straight leg raise (SLR) of > 91° and age of < 40 years [25].

A study by Areeudomwong et al. [9] reported a combination of four tests for classifying lumbar instability as follows: apprehension sign, instability catch sign, painful catch sign and prone instability test. When at least 3/4 of these tests were positive, a sensitivity of 47.8% and specificity of 91.7% were recorded [9].

However, due to the limited evidence available and the fact that previous research is based on patients from hospital settings with a narrow age range, such as 45-67 years [21], 56-80 years [23], 43-68 years [24] and 41-59 years [9], the generalizability of these results to people with CLBP may be limited. Calculating measures of effect from predicted probabilities following logistic regression is an appropriate method to infer the overall source population from which the study sample was drawn [26]; however, this has not been done to date.

The purpose of the present study was to examine the relationship between 14 clinical tests, obtained from prior studies [7, 21–25] and X-ray findings for patients with CLBP in community-based health settings, in order to establish the probability of existing lumbar instability across a wide age range (20–60 years). This could inform treatment decision-making without having to rely on radiography, as access to X-rays may be limited. In addition, this test can provide supporting information to justify the referral of patients to X-ray services for further examination.

## Materials and methods

### Methods

The study protocol for human research was approved by The Human Research Ethics Committee of Khon Kaen University, Thailand according to the declaration of Helsinki (HE 602379). The study was prospectively registered at Thai Clinical Trials Registry (TCTR 20180820001).

### Participants

According to Long [27], and Voorhis and Morgan [28], a minimum of 10 participants per parameter is a sufficient number for a logistic regression model. As the current study included 14 clinical tests, the sample size target was 140 CLBP participants aged 20-60 years with or without pain radiation into the lower extremities that lasted > 3 months.

Potential participants were recruited via posters and social media advertisements in a community-based setting. The selection of participants was undertaken using a convenience sampling approach. The exclusion criteria included the following; contraindication to X-ray assessment (e.g., pregnancy), previous lumbar surgery, serious spinal pathology (e.g., cauda equina syndrome, malignancy, vertebral fracture and infection), scoliosis, neurological deficit and spondylolisthesis [12, 29].

### Procedure

Prior to the main data collection, 16 participants with CLBP were assessed by a physical therapist who conducted the 14 clinical assessments to evaluate intra-rater reliability. The main study participants were assessed at 2 sessions.

During the first session, participants signed the consent form and the investigators collected their demographic data [age, sex, Roland Morris Disability Questionnaire (RMDQ) [30], body mass index and pain information]. Participants were asked about pain duration, pain radiation, whether they were currently undergoing a pain episode, and the average pain score in the last 24 h; 14 clinical assessments were then conducted (Additional file 1).

During the second session, participants were evaluated by an orthopaedic surgeon who ordered an X-ray assessment. Six views were used for plain radiographs; anteroposterior, lateral, two oblique, lateral flexion, and lateral extension with participants positioned in side-lying. The X-ray procedure was completed by a radiologist. The lateral flexion-extension radiographs were then inspected for lumbar instability by a trained observer.

### X-ray measurement technique

**Fig. 1:**
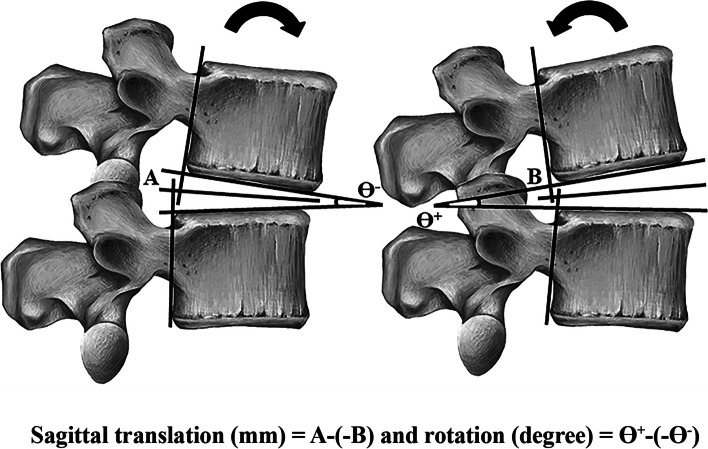
Measurement technique of lumbar instability [12]. Angulation: Two straight lines are draw along the inferior endplate of the upper vertebra and the superior endplate of the lower vertebra. The intersection of these two lines is angulation in flexion (Ɵ^−^) and in extension (Ɵ^+^). The difference of intervertebral angles between Ɵ^−^ and Ɵ^+^ is amount of rotational instability. Translation: The straight line is drawn bisect the angulation in flexion (Ɵ^−^) and in extension (Ɵ^+^). The difference between the two distances from flexion (A) and extension (B) is amount of translation

All X-ray images for each participant were read by a trained observer who was blinded to the clinical test results. Imaging-related findings have been previously used to confirm the presence of lumbar instability [7, 12, 21–24]. The amount of sagittal plane translation and rotation occurring at individual spinal motion segments between L_1-2_ and L_5_-S_1_ was calculated from the X-ray films (Fig. 1) [12].

The reference value of lumbar instability was defined as a translation of > 4.5 mm at L_1-2_ to L_5_-S_1_ and rotation of > 15° at L_1-2_, L_2-3_ and L_3-4_, of > 20° at L_4-5_, and of > 25° at L_5_-S_1_ [31]. The participants was considered to have lumbar instability when (i) two segments exhibited rotation or translation instability, or when (ii) one segment exhibited both translation and rotation instability [7, 12].

### Data analysis

Descriptive statistics were calculated and sequestered into groups of participants with and without a diagnosis of lumbar instability, as diagnosed using X-ray. Continuous variables were compared using independent t-tests and categorical variables using Pearson’s χ^2^ tests. A significance level of *P* < 0.05 was used for all variables.

The reliability of X-rays and clinical examination variables was examined. Kappa coefficients were used for dichotomous variables and intraclass correlation coefficients (ICC) (3, 1) were used for continuous variables.

Univariate and multivariate logistic regression were used to analyse the predictive test of lumbar instability. The clinical tests that reached a *P* < 0.2 in the univariate analysis were considered to be associated with lumbar instability and were entered into the first model of multivariate regression analysis. *P* < 0.2 was selected in order to prevent missing any clinical tests that might be associated with lumbar instability [32, 33].

Backward stepwise elimination was used to build the model. During each of the backward stepwise techniques, pseudo-R-squared and goodness of fit test [Akaike information criterion (AIC), Bayes information criterion and area under the curve (AUC)] were conducted to compare the performance of each model. The model with the lowest AIC value was considered to be the best one [34]. Moreover, *P* < 0.05 was considered significant in the final model, and the variables retained were used to develop the probability to diagnose existing lumbar instability. Data analysis was conducted using STATA 10.0 (StataCorp LP; College Station, TX, USA).

## Results

**Fig. 2:**
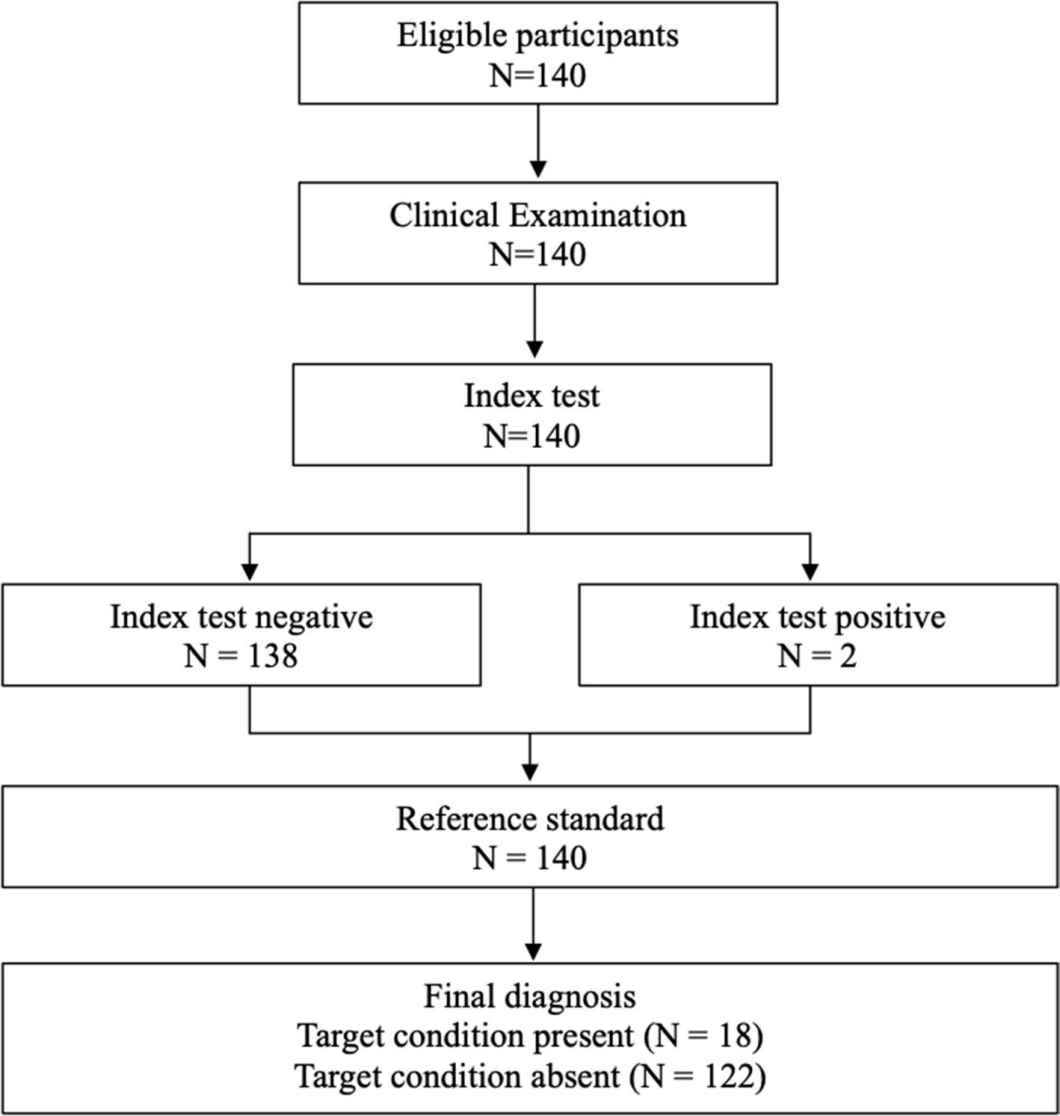
STARD flow diagram

A total of 140 participants with CLBP were included in the present study; a STARD flow chart can be seen in Fig. 2.

The patients’ demographic characteristics are described in Table 1; of the 140 participants, 12.86% had lumbar instability and 87.14% did not. The majority of variables were not different between the two groups. Only the RMDQ was significantly (*P* = 0.04) higher in the lumbar instability group. The mean difference in the RMDQ score was 2.58, which was less than the minimal clinical important difference of 3.5 [35].

**Table 1:** Demographic characteristics of the 140 participants with and without lumbar instability

	Without lumbar instability (*n* = 122)		Lumbar instability (*n* = 18)		***P***- value
	n(%)	Mean ± SD	n(%)	Mean ± SD	
**Age** (years)		35.80 ± 12.35		37.00 ± 12.32	0.70
**Sex**					
• Male	49(40.16)		5(27.78)		0.31
• Female	73(59.84)		13(72.22)		
**BMI**		22.29 ± 2.37		21.97 ± 3.12	0.61
**RMDQ** score		6.08 ± 4.90		8.67 ± 5.41	0.04*
**Pain duration** (months)		25.71 ± 25.25		38.33 ± 48.65	0.09
**Pain episode**					
• First	63(51.64)		13(72.22)		0.10
• Recurrent	59(48.36)		5(27.78)		
**Pain radiation**					
• Yes	64(52.46)		11(61.11)		0.49
• No	58(47.54)		7(38.89)		
**Pain score** (NRS)		4.52 ± 1.46		4.94 ± 1.35	0.24

*Abbreviations*: *SD* Standard deviation, *BMI* Body Mass Index, *RMDQ* Roland-Morris Disability Questionnaire, *NRS* Numerical Rating Scale, *n* number

*P-value < 0.05

A total of 18 (12.86%) participants had lumbar instability. Their average age was 37.0 ± 12.32 years (range, 20-53 years), with 13 women and 5 men. When classified into age groups, 6 (33%) were aged 20-29 years, 4 (22%) 30-39 years, 2 (11%) 40-49 years and 6 (33%) 50-59 years.

Among of the 47 segments, the most commonly recorded levels of instability were L_4-5_ (*n* = 20; 42.55% of segments), L_5_-S_1_ (*n* = 13; 27.66% of segments), L_2-3_ (*n* = 7; 14.89% of segments), L_3-4_ (*n* = 6; 12.77% of segments) and L_1-2_ (*n* = 1; 2.13% of segments).

**Fig. 3:**
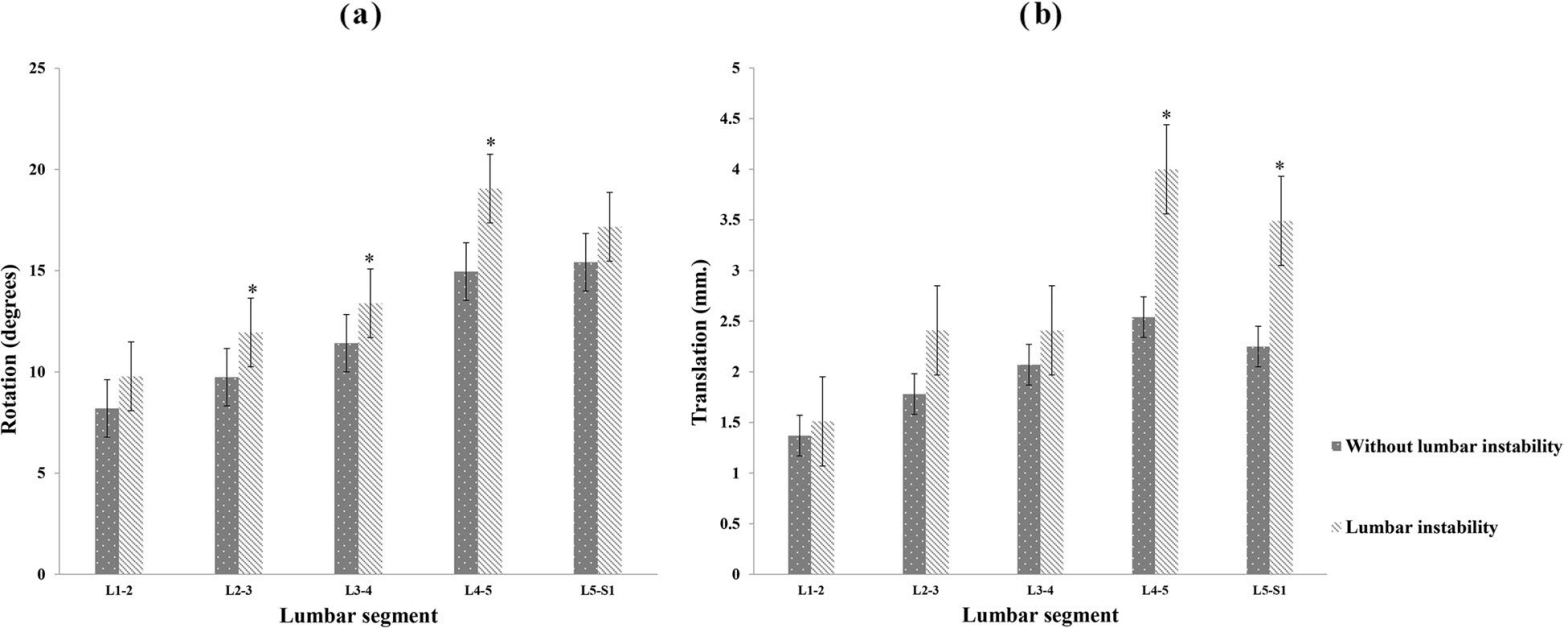
Comparison of sagittal rotation (**a**) and tanslation (**b**) in participant with and without lumbar instability

Sagittal rotation and translation were compared between participants with and without lumbar instability (Fig. 3). Rotation instability was significantly greater at the L_2-3_, L_3-4_ and L_4-5_ levels (*P* < 0.05) in participants with lumbar instability. For translation, this was significantly greater at L_4-5_ and L_5_-S_1_ levels (*P* < 0.05) in the lumbar instability group.

The ICC of the X-ray measurement technique was 0.83 (95% CI: 0.46-0.96) for rotation and 0.93 (95% CI: 0.74-0.98) for translation, suggesting a good measurement consistency. The Kappa coefficient of the 14 clinical tests ranged from moderate to excellent (Table 2). Three tests demonstrated moderate reliability [‘passive physiological intervertebral movements (PPIVMs) (flexion)’, ‘passive lumbar extension test’ and ‘aberrant motion test’]. Four tests demonstrated good reliability [‘passive accessory intervertebral movements’ (PAIVMs), ‘painful catch sign’, ‘posterior shear test’ and ‘interspinous gap change’]. The ‘total trunk extension’, ‘lumbar flexion’, ‘Beighton’s scale’, ‘PPIVMs (extension)’, ‘prone instability test’, ‘sit to stand’ and ‘average SLR’ tests exhibited excellent reliability [36, 37].

**Table 2:** Reliability coefficients of 14 clinical examinations (*n* = 16)

Clinical tests	Percent agreement	Kappa (95%CI)	Interpretation
1. Total trunk extension > 26°	100	1.00 (1.00 to 1.00)	Excellent
2. Lumbar flexion > 53°	100	1.00 (1.00 to 1.00)	Excellent
3. Average SLR > 91°	100	1.00 (1.00 to 1.00)	Excellent
4. Beighton’s scale	100	1.00 (1.00 to 1.00)	Excellent
5. PPIVMs (flexion)	75.00	0.50 (0.02 to 0.98)	Moderate
6. PPIVMs (extension)	91.67	0.83 (0.53 to 1.00)	Excellent
7. PAIVMs	88.33	0.67 (0.25 to 1.00)	Good
8. Passive lumbar extension	88.33	0.56 (0.01 to 1.00)	Moderate
9. Painful catch sign	91.67	0.63 (−0.03 to 1.00)	Good
10. Posterior shear test	91.67	0.63 (−0.03 to 1.00)	Good
11. Prone instability test	91.67	0.82 (0.50 to 1.00)	Excellent
12. Aberrant motion test	83.33	0.56 (0.01 to 1.00)	Moderate
13. Interspinous gap change	83.33	0.63 (0.16 to 1.00)	Good
14. Sit to stand	100	1.00 (1.00 to 1.00)	Excellent

*Abbreviations*: *PPIVMs* Passive Physiological Intervertebral Movements, *PAIVMs* Passive Accessory Intervertebral Movements, *CI* Confidence interval, *SLR* Straight leg raise

The univariate regression analyses results are presented in Table 3. The interspinous gap change, PAIVMs, PPIVMs (flexion), PPIVMs (extension), painful catch sign and posterior shear tests reached a *P* < 0.2, which suggested a good to excellent reliability (Table 2).

**Table 3:** Univariate analyses

Clinical test	Crude odds ratio	95%CI odd ratio	***P***-value
1. Total trunk extension > 26°	0.54	0.18 to 1.60	0.26
2. Lumbar flexion > 53°	1.65	0.59 to 4.60	0.34
3. Average SLR > 91°	0.63	0.19 to 2.05	0.44
4. Beighton’s score > 2°	1.74	0.18 to 16.46	0.63
5. PPIVMs (flexion)	2.07	0.76 to 5.61	0.16*
6. PPIVMs (extension)	4.68	1.03 to 21.28	0.05*
7. PAIVMs	9.25	1.19 to 71.93	0.03*
8. Passive lumbar extension	1.90	0.70 to 5.16	0.21
9. Painful catch sign	3.22	0.70 to 5.16	0.13*
10. Posterior shear test	2.94	1.07 to 8.06	0.04*
11. Prone instability test	0.95	0.34 to 2.61	0.92
12. Aberrant motions test	1.04	0.32 to 3.42	0.95
13. Interspinous gap change	2.69	0.97 to 7.46	0.06*
14. Sit to stand	0.59	< 0.001 to 4.07	0.65

*P-value < 0.2: significant risk factors from univariate analyses

Six tests were therefore selected for the initial model of multivariate logistic regression. The backward stepwise technique was used to eliminate one clinical test per model. Pseudo-R-squared and four types of goodness of fit test were performed for each model (Table 4). The final model underwent the interspinous gap change, PAIVMs and posterior shear tests, since they had the lowest AIC and a good AUC. Moreover, the Hosmer-Lemeshow test reached a *P* = 0.33, which suggested that the model fit the data reasonably well [38].

**Table 4:** The characteristic of each model during backward stepwise technique

	Pseudo-R-squared	Hosmer-Lemeshow	AIC	BIC	AUC
Initial model					
1.Interspinous gap change	0.23	0.12	98.98	122.52	0.83
2. PAIVMs					
3. PPIVMs in flexion					
4. PPIVMs in extension					
5. Painful catch sign					
6. Posterior shear test					
Excluded painful catch sign	0.21	0.38	96.41	114.06	0.83
Excluded PPIVMs in extension	0.20	0.09	96.22	110.93	0.81
Exclude PPIVMs in flexion					
**1. Interspinous gap change**	**0.18**	**0.33**	**95.72**	**107.48**	**0.78**
**2. PAIVMs**					
**3. Posterior shear test**					

*Abbreviations*: *AIC* Akaike information criterion, *BIC* Bayes information criterion, *AUC* Area Under Curve

To confirm the combination of the three clinical tests as the final model, multivariate analysis was performed with *P* < 0.05 (Table 5).

**Table 5:** Multivariate analyses

Clinical test	Adjust odds ratio	95%CI	***P***-value
1. Interspinous gap change	5.75	1.58 to 20.99	0.008*
2. PAIVMs	13.90	1.55 to 124.30	0.02*
6. Posterior shear test	5.42	1.53 to 19.17	0.009*

*P-value < 0.05: significant risk factors from multivariate analyses

Based on the final model of the multivariate logistic regression analysis, the probability of having lumbar instability can be calculated as $\mathrm{Prob}\left(\mathrm{lumbar}\ \mathrm{instability}=1\right)=\frac{1}{1+{\mathrm{e}}^{-\left(-5.37+1.75\left(\mathrm{X}1\right)+2.63\left(\mathrm{X}2\right)+1.69\left(\mathrm{X}3\right)\right)}}$. The X1, X2 and X3 were replaced with 0 (negative result) and 1 (positive result) in the interspinous gap change, PAIVMs and posterior shear test. When the three tests were positive, the predictive model produced a 67% probability of patients with CLBP having lumbar instability. At this predictive threshold, sensitivity, specificity, +LR, and -LR were 5.56% (95% CI: 0.14-27.30), 99.18% (95% CI: 95.5-100), 6.78 (95% CI: 0.44-104) and 0.95 (95% CI: 0.85-1.07), respectively.

## Discussion

To the best of our knowledge, this was the first study to investigate the predictive probability of clinical tests in patients with lumbar instability. When used in combination, a positive result from three clinical tests (interspinous gap change, PAIVMs and posterior shear tests) indicated a probability of 67% for the presence of lumbar instability in patients.

Study participants with lumbar instability were similar to clinical populations in that they were mostly middle-aged females with instability at the L_4-5_ level [7, 21–24]. However, when considering the age group of patients with lumbar instability, a similar number of patients with lumbar instability were aged 20-29 and 50-59 years. This result suggested that physical therapists should also look out for lumbar instability in young patients with CLBP.

In the present study, the percentage (12.86%) of CLBP patients with lumbar instability in the study population was lower than previously reported (57%) [7]. This may have been due to the wide age range (20-60 years) of participants in this, compared to previous, studies, since lumbar instability has been found to be more prevalent in individuals aged > 40 years [7, 21–24]. In addition, the lumbar instability criteria in the present study were more comprehensive than those of earlier studies, in an attempt to minimize the number of false-positive X-rays reported in earlier studies [7]. Finally, this study included individuals with CLBP drawn from a community sample, as opposed to the hospital-based samples of previous studies [7, 21–24].

The present study reported that patients with lumbar instability typically had a longer duration of symptoms and a higher frequency of pain radiation, compared with those without lumbar instability. However, whilst there was a statistical difference in the RMDQ between the groups, this was less than the minimal clinical important difference. The disability score of participants with lumbar instability was higher in this study than that in earlier studies [7, 23]. As reported by previous studies, instability was more frequently observed at the L_4-5_ level, which was due to the orientation of the zygapophyseal joint being inclined in the sagittal plane [21, 24].

In the present study, a full flexion-extension X-ray in a side-lying position was used to avoid the influence of muscle bracing and aggravation of the patient’s pain during movement while standing [12, 39–41]. These postures produced a significantly higher rotation (at the L_2-3_, L_3-4_ and L_4-5_ levels) and translation (at the L_4-5_ and L_5_-S_1_ levels) in participants with lumbar instability, compared with those without lumbar instability. These results highlighted the possibility that excessive lumbar movement was not only induced in standing and seated flexion-extension, but also in lateral side-lying flexion-extension, which was consistent with previous findings [12, 39–41].

Regression analysis showed that the three clinical tests in the final model fit the data well and had a satisfactory reliability range (from good to moderate). These findings confirm that these three tests related to test the passive stability subsystem. It was shown herein that, when all three tests were positive, the probability of having lumbar instability was 67%, with the sensitivity, specificity, +LR and -LR at 5.56, 99.18%, 6.78 and 0.95, respectively. Thus, the combination of these three tests can predict lumbar instability with a high specificity (99%) and a moderate +LR (6.78) [42]. Furthermore, the final model had an AUC of 0.78, which indicated an adequate discriminative ability between patients with CLBP who have and do not have lumbar instability [38].

A high sensitivity (82.2%), moderate specificity (60.7%), and low +LR (2.1) and -LR (0.3) have been reported for the interspinous gap change test, which was developed by Ahn and Jung [24]. The interspinous gap change test was undertaken in a standing position with lumbar spine flexion-extension performed passively through the hip joint. Excessive movements at the end of lumbar spine flexion-extension were due to passive subsystem dysfunction, leading to an inability to control spine movement within the normal range.

PAIVMs are commonly utilized when physical therapists identify movement abnormalities in the lumbar spine, be that joint hyper- or hypomobility [22]. This technique has been assessed during the absence of muscle activation, with the passive stability of the lumbar spine being the target for the test [43]. In the present study, increasing movement was observed at the degenerative region, suggesting that the restraining structures cannot limit the displacement of that lumbar segment. Abbott et al. [22] reported that PAIVMs had a high specificity (89%) and low sensitivity (29%), which was consistent with the results of Fritz et al. [7], who reported a specificity of 81% and sensitivity of 46%.

Fritz et al. (2005) reported that the posterior shear test has a low diagnostic accuracy [7], which was inconsistent with the present findings. Although both studies used the same test protocol, it is unclear why the result was different. One possible reason for this could be a variation in the force applied by the examiner to the participant’s abdomen [7]. The examiner of the current study may have applied greater force compared to the examiner used in the study by Fritz et al. [7], leading to a positive posterior shear test result in the present study.

The three predictive tests in this study had a higher specificity when used as indicative tests of passive subsystem dysfunction, compared with previous studies [7, 22, 24]. Furthermore, to the best of our knowledge, the present study was the first to use predictive probability, AIC and AUC to support the test results. These tests could provide valuable information for physical therapists during the clinical assessment of patients with CLPB suspected of having lumbar instability. However, while the combination of the three tests exhibited a satisfactory reliability and high specificity, which would enable the physical therapist to identify patients who are very likely to have lumbar instability, their low sensitivity may not help rule out the diagnosis of lumbar instability.

Several clinical tests, such as aberrant motion, sit to stand, lumbar flexion and prone instability tests, were not found to be associated with instability, as expected. As all the tests were active movement tests, it can be speculated that, when there is passive subsystem dysfunction, the remaining subsystems (active and neural control) provide a compensatory change, thus influencing spinal stability [3, 44]. Therefore, patients may not exhibit instability signs during these tests.

The findings of the present study can prove useful for clinicians. However, the study was not without its limitations. First, the prevalence of lumbar instability was smaller than expected. Although, the sample size was calculated using the rule of thumb, had a power of 0.98 and was considered acceptable [45] a sample size that includes a higher percentage of participants with lumbar instability is recommended in further research. Secondly, pain in full flexion-extension may have led to a lower amount of translation and rotation instability than the actual amount experienced by the patients, although their position was adjusted by the radiologist. Thirdly, the current study design may not be recommended for the establishment of predictive tests; a longitudinal study design may instead help obtain more accurate results.

## Conclusion

To the best of our knowledge, this was the first study that presented the probability of the three tests combination to predict lumbar instability in community-based patients with CLBP across a wide age range. Three clinical tests that assess the passive stability subsystem were found to predict lumbar instability with a satisfactory reliability. These tests were the interspinous gap change, PAIVMs and posterior shear test. When patients with CLBP have positive results on these three tests, there is a 67% probability that they suffer from lumbar instability.

## Supplementary Information

**Additional file 1.** Methods of the 14 clinical examination.

## Data Availability

The data will be available for anyone who wishes to access them for any purpose. The data will be accessible from immediately following publication to 6 months after publication, and contact should be made via the Corresponding author rungthiprt@gmail.com.

## Abbreviations

CLBP: Chronic low back pain
RMDQ: Roland Morris Disability Questionnaire
ICC: Intraclass correlation coefficients
-LR: Negative likelihood ratio
+LR: Positive likelihood ratio
AIC: Akaike information criterion
BIC: Bayes information criterion
AUC: Area under the curve
PAIVMs: Passive accessory intervertebral movement tests
PPIVMs: Passive physiological intervertebral movement tests
SLR: Straight leg raise
