# Supplementary material for: A diagnostic tool for people with lumbar instability: a criterion-related validity study
Source: BMC Musculoskelet Disord. 2021 Nov 23;22:976. doi: 10.1186/s12891-021-04854-w (PMC8609735; doi:10.1186/s12891-021-04854-w)
Supplement: Supplementary file 1 — Additional file 1. Methods of the 14 clinical examination. [file 12891_2021_4854_MOESM1_ESM.docx]

| **Additional file** Methods of the 14 clinical examination | |
| --- | --- |
| **Tests** | **Methods and interpretation** |
| **1. Painful catch sign test** | The painful catch sign test begins in a supine lying position. The process is as follows:   1. The participants are asked to lift their lower extremities while keeping their knees extended from the examination table. 2. Then, they return their lower extremities slowly down to the examination table.   Where the participant’s lower extremities fall suddenly to the examination table because of sudden low back pain, this is judged to be a **positive** result. |
| **2. Aberrant motion test** | The aberrant motion test begins in a standing position. The participants are required to flex their trunk forward as far as possible and return to an upright position while the examiner observes the following abnormalities:  1) Painful arc in flexion  2) Painful arc on return to erect position  3) Gower sign or thigh climbing: on attempting to return to the erect position, participants push on their thighs or other surface for support.  4) Instability catch: participants are unable to return to an upright position and/or acceleration or deceleration of trunk movement due to sudden low back pain.  5) Reversal of lumbopelvic rhythm: before returning to the erect position, the participants bend their knees and shift the pelvis anteriorly.  Where at least one of the abnormal movement patterns is present, the test is interpreted as a **positive** result. |
| **3. Prone instability test** | The prone instability test requires the participant to lie prone on an examination table with his/her trunk resting on the examination table, their hips flexed over the edge of the table, and their feet resting on the floor. The process is as follows;  1) With the feet on the floor (position 1): the examiner applies a posterior-anterior (PA) force over each of lumbar spinous process. If participant reports an increased pain with a PA force, position 2 test is undertaken.  2) The participant lifts their feet off the floor (they may hold the examination table to maintain the position) (position 2): A PA force is repeated at the pain level of position 1.  If pain in position 2 decreases compared to position 1, the test is judged as a **positive** result. |
| **4. Posterior shear test** | The posterior shear test requires the participant to stand with his/her hands across located on their lower abdomen. The process is as follows:  1) The examiner stands by side of the participant and places one hand on the participant’s cross hands. The examiner places the heel of their other hand on the participant’s pelvis.  2) The examiner produces a posterior shear force through the participant’s abdomen and an anterior stabilizing force with the other hand.  3) The test is repeated at each lumbar level. |
|  | A **positive** result occurs when pain is provoked in the lumbar region. |
| **5. PAIVMs** | The passive accessory intervertebral movements (PAIVMs) begin with the participant in prone. The process as follows:  1) The examiner places hypothenar eminence on the spinous process and produces a PA force. Pain is recorded at each level.  2) The test is repeated at each lumbar level.  A **positive** result occurs when mobility is judged as hypermobile and there is increased pain at any lumbar segment. |
| **6. PPIVMs in flexion and 7. extension** | The passive physiological intervertebral movements (PPIVMs) begin with the participant in a side-lying position. The hips of the participant are flexed at 90º. The process is as follows:   1. One hand of the examiner palpates between the spinous process of adjacent vertebrae. 2. The examiner’s other hand produces flexion and extension through the participant’s femurs.   A **positive** test is identified by the detection of hypermobility during flexion and extension. |
| **8. Beighton’s hypermobile scale** | Beighton’s hypermobility scale is assessed with the nine-point scale. The 9 points are made up of the following;   1. Ability to flex the trunk and place hands flat on the floor with knees extended. 2. Elbow hyperextension >10˚ 3. Knee hyperextension >10˚ 4. Fifth finger hyperextension >90˚ |
|  | 1. Thumb abduction to contact the forearm   The **total score more than 2** is interpreted as a **positive** test. |
| **9. Lumbar flexion test** | The range of motion is measured with two inclinometers. The assessment is as follows:  1) The participant is in the erect position. Inclinometers are placed at T_12_-L_1_ (A) and S_2_ (B) by the examiner.  2) The participant is asked to bend his/her trunk as far as possible keeping knees straight; flexion angle is recorded at T_12-L1_ (C) and S_2_ (D).  3) The degrees at point C-A=total lumbar flexion, and D-B=sacral flexion.  4) The difference between C and D is lumbar flexion.  The **degree of lumbar flexion more than 53˚** is interpreted as a **positive** test. |
| **10. Passive lumbar extension test** | The participant is in a prone position. The assessment is as follows:   1. Both the participant’s legs are passively raised by the examiner to a height of 30 cm from examiner table with knees extended. 2. Then, the examiner gently pulls the participant’s lower extremities.   The test is interpreted as a **positive** result when the participant complains of pain in the lumbar region and such pain reduces when participant returns to the starting position. |
| **11. Sit to stand test** | The participant is asked to report pain that occurs immediately or within 2 to 3 minutes of sitting down and totally or partially relieved by standing up from sitting.  The test is interpreted as a **positive** result when the participant reports pain as mentioned. |
| **12. Total extension test** | The range of motion is measured with a single inclinometer. The assessment is as follows:  1) The participant is in the erect position. Inclinometer is placed at T_12_-L_1_ (A) by the examiner.  2) The participant is asked to extend their trunk, bending back as far as possible while keeping his/her knees straight; the extension angle is recorded at T_12-_L_1_ (B).  3) The degrees at point B-A=total lumbar extension.  The **degree of a total extension** **more than 26˚** is interpreted as a **positive** test. |
| **13. Interspinous gap change during flexion-extension** | The interspinous gap change during flexion-extension is performed in a standing position. The procedure is as follows:  1) The participant is asked to flex his/her back and place both hands on the examination table. The examiner places the pad of their thumb on the upper and lower interspinous space. If an interspinous space is wider than the adjacent interspinous spaces, it is suspected of being an unstable level.  2) Then, the participant is asked to push his/her pelvis toward the examination table with both hands on the table reproducing lumbar extension.  3) During extension, the examiner evaluates the change in the suspected unstable level. |
|  | The test is considered **positive** if an interspinous space of suspected level has a wider superior-inferior or AP gap, or becomes abruptly narrow during the flexion-extension motion compared with adjacent interspinous spaces. |
| **14. Average SLR >91°** | The patient is supine. The inclinometer is positioned on the tibial crest just below the tibial tubercle. The leg is raised passively by the examiner, whose other hand maintains the knee in extension.  **Positive** test is ROM more than 91 degrees. |
